# Identifying Genes Devoted to the Cell Death Process in the Gene Regulatory Network of *Ustilago maydis*

**DOI:** 10.3389/fmicb.2021.680290

**Published:** 2021-05-21

**Authors:** Cinthia V. Soberanes-Gutiérrez, Ernesto Pérez-Rueda, José Ruíz-Herrera, Edgardo Galán-Vásquez

**Affiliations:** ^1^Departamento de Ingeniería Genética, Unidad Irapuato, Centro de Investigación y de Estudios Avanzados del Instituto Politécnico Nacional, Irapuato, Mexico; ^2^Laboratorio de Ciencias Agrogenómicas, de la Escuela Nacional de Estudios Superiores Unidad León, Universidad Nacional Autónoma de México, León, Mexico; ^3^Unidad Académica Yucatán, Instituto de Investigaciones en Matemáticas Aplicadas y en Sistemas, Universidad Nacional Autónoma de México, Mérida, Mexico; ^4^Departamento de Ingeniería de Sistemas Computacionales y Automatización, Instituto de Investigación en Matemáticas Aplicadas y en Sistemas, Universidad Nacional Autónoma de México - Ciudad Universitaria, Mexico City, Mexico

**Keywords:** *U. maydis*, cell death, regulatory networks, transcription factors, apoptosis, necrosis, autophagy

## Abstract

Cell death is a process that can be divided into three morphological patterns: apoptosis, autophagy and necrosis. In fungi, cell death is induced in response to intracellular and extracellular perturbations, such as plant defense molecules, toxins and fungicides, among others. *Ustilago maydis* is a dimorphic fungus used as a model for pathogenic fungi of animals, including humans, and plants. Here, we reconstructed the transcriptional regulatory network of *U. maydis*, through homology inferences by using as templates the well-known gene regulatory networks (GRNs) of *Saccharomyces cerevisiae*, *Aspergillus nidulans* and *Neurospora crassa.* Based on this GRN, we identified transcription factors (TFs) as hubs and functional modules and calculated diverse topological metrics. In addition, we analyzed exhaustively the module related to cell death, with 60 TFs and 108 genes, where diverse cell proliferation, mating-type switching and meiosis, among other functions, were identified. To determine the role of some of these genes, we selected a set of 11 genes for expression analysis by qRT-PCR (*sin3, rlm1, aif1, tdh3* [isoform A], *tdh3* [isoform B], *ald4, mca1, nuc1, tor1, ras1*, and *atg8*) whose homologues in other fungi have been described as central in cell death. These genes were identified as downregulated at 72 h, in agreement with the beginning of the cell death process. Our results can serve as the basis for the study of transcriptional regulation, not only of the cell death process but also of all the cellular processes of *U. maydis*.

## Introduction

Cell death is a conserved and essential process which confers an adaptive advantage to the organisms, and manifests with macroscopic morphological alterations. Cell death is classified into three main different categories: (1) apoptosis or type I cell death, (2) autophagy or type II cell death, and (3) necrosis or type III cell death (Falcone and Mazzoni, 2016; Galluzzi et al., 2018).

Apoptosis occurs when DNA damage is irreparable, and it is characterized by morphological changes, such as cellular shrinkage, chromatin condensation (pyknosis), nuclear fragmentation (karyorrhexis), loss of adhesion to neighbors cells or to extracellular matrix and dynamic membrane blebbing (Elmore, 2007). At a biochemical level, the chromosomal DNA is cleavage into internucleosomal fragments, and membrane phosphatidylserine externalization and a number of intracellular substrates are breakdown by specific proteases, finishing with the formation of apoptotic bodies (small vesicles) to be posteriorly degraded within vacuoles (Matsuyama et al., 1999; Wloch-Salamon and Bem, 2013).

Autophagy is characterized by accumulation of double-membraned vesicles termed autophagosomes, and bulk degradation of long-lived proteins. In general, there is delivery of the organelles or cytoplasmic components to vacuole (autophagic vacuoles) and culminating with phagocytic uptake and consequent vacuolar degradation, allowing a cell to monitor membrane continuity or integrity (Wloch-Salamon and Bem, 2013).

Finally, necrosis is involved into the preservation of tissue homeostasis, to eliminate damaged cells. In this process, there is a rapid loss of plasma membrane integrity, mitochondrial dysfunction, and organelle swelling (Hitomi et al., 2008).

In fungi, most studies on cell death are focused on *Saccharomyces cerevisiae*, *Schizosaccharomyces pombe* and *Candida albicans* (Ramsdale, 2008; Gonçalves et al., 2017). This process is induced in response to exogenous components such as plant defense molecules, toxins and fungicides, during non-self-recognition, and heterokaryon incompatibility, and during developmental programs throughout the fungal life cycle, including morphogenesis associated with plant infection (Ramsdale, 2008; Gonçalves et al., 2017).

*Ustilago maydis* is a Basidiomycota biotrophic fungus which causes common smut in corn (*Zea mays*) and its progenitor, the teozintle (Z. *mays* subsp. *parviglumis*) (Agrios, 2005). *U. maydis* causes infection symptoms such as chlorosis, increased anthocyanin biosynthesis and large galls in the aerial parts of the plant (Lanver et al., 2017). To complete its life cycle, this fungus requires the plant (García-Muse et al., 2003), and it is an excellent model for dimorphism and virulence.

In a previous work, we identified that in artificial media, *U. maydis* loses its viability in a noticeably shorter time compared with *S. cerevisiae*, *Yarrowia lipolytica*, and *Sporisorium reilianum*. Addition of curcumin or metformin increased the half-life of the fungus (Soberanes-Gutiérrez et al., 2020). These findings indicate that there are differences in the process of cell death of these yeasts compared with other fungi, and that the shortness of *U. maydis* lifespan is not related to its pathogenicity, or to its dimorphism. For that reason, we are interested in analyzing the genes involved in cell death of *U. maydis.*

In this context, the gene regulatory networks (GRNs) control the cellular processes of the organisms. GRN can be conceptualized in a graph which consists of two main components: nodes that represent the regulatory proteins (TFs) and target genes (TGs); and edges that represent the physical and/or regulatory relationships between the nodes (Babu et al., 2004; Peter and Davidson, 2015). Only few GRNs have been experimentally characterized for fungal organisms, such as *S. cerevisiae* (Monteiro et al., 2020), *Aspergillus nidulans* and *Neurospora crassa* (Hu et al., 2018). For this reason, homology-based approaches tend to be a solution for the study of GRNs in other lesser-known organisms (Galán-Vásquez et al., 2016; Lenz et al., 2020).

An analysis of the regulation of the cell death process in *U. maydis* will provide clues to understand the differences with other fungal systems, focusing on the study of its cell death mechanism. To this end, we inferred its gene regulatory network (GRN) through homology relationships from three fungal genomes with GRNs experimentally described. From this reconstruction, we identified and analyzed the genes associated with the cell death module, from which a set of 11 genes were posteriorly analyzed by qRT-PCR.

## Data and Methodology

### Reconstruction of the GRN

The GRN is a collection of regulatory interactions that can be represented in a graph as *G = (V,A)*, where *V* is a set of vertices that correspond to genes or proteins in the network and *A* is a set of edges, and every edge *(u,v)* connects two vertices, *u* and *v*. The network can be directed, *i.e.*, the interaction goes from *u* (the tail) to *v* (the head), or undirected, where there is no direction of the interaction between any or two vertices.

To reconstruct the GRN in an organism with no regulatory interactions experimentally described, a comparative genomics approach can be used. To this end, the GRN from a model organism can be used as a template to export interactions in the organism of interest. This inference is based on the assumption that orthologous TFs generally regulate the expression of orthologous TGs (Yu et al., 2004; Galán-Vásquez et al., 2016). In this work, three templates were considered for the inference of the GRN of *U. maydis*. GRN of *S. cerevisiae* was obtained from YEASTRACT database, only the interactions with experimental evidence were considered, which is composed of 6,709 nodes and 179,601 interactions (Monteiro et al., 2020); *A. nidulans* with 5,969 nodes and 10,018 regulatory interactions; and *N. crassa* with 7,446 nodes and 20,499 regulatory interactions (Hu et al., 2018).

To identify orthologous proteins between the *U. maydis* proteome and the proteomes of *A. nidulans, N. crassa*, and *S. cerevisiae*, we used the program ProteinOrtho (V6.0.15) (Lechner et al., 2011), with the following parameters: *E*-value of 0.01, a sequence coverage of 50%, and minimal percent identity of best blast hits in 30%, except for the report of singleton genes without any hit. OrthoVenn2 was used to identify orthologous clusters in the four proteomes, and to perform a functional enrichment analysis for each cluster, we used the *E*-value cutoff of 0.01 for all-to-all protein similarity comparisons, an inflation value of 1.5 for the orthologous clustering using Markov Cluster Algorithm. The enrichment analysis was considered significant with a *P*-value less than 0.05 (Xu et al., 2019). Our reconstruction approach considers that if the sequences corresponding to TFs and TGs are conserved, then regulatory interactions are also conserved (Supplementary Table 1).

### TF Identification

To assess the diversity of TFs, protein sequences of whole proteomes were used to search TF domains using InterProScan (v5.25-64.0) (Jones et al., 2014) and hmmsearch (v3.1b2) (Potter et al., 2018). InterProScan was used to map Interpro families and domains, while hmmscan was used to identify PFAM domains over the pfam database (v31.0-2017-02) using default parameters (El-Gebali et al., 2019; Supplementary Table 2).

### Network Structural Analysis

To determine the structure of the reconstructed network the following topological metrics were calculated: node degree, clustering coefficient, connectivity, hubs, and communities (Junker and Schreiber, 2011). The node degree (*K*) corresponds to the number of edges that it has with other nodes. In directed networks as GRN, input degree (*Kin*) is the number of arrows that enter to node, which corresponds to the TFs that affect a TG; output degree (*Kout*) is the number of arrows that leave a node, and corresponds to the number of TGs by which a TF is regulated (Barabási and Oltvai, 2004).

The connectivity is the association between each pair of nodes, which can be via a direct or indirect edge through intermediate connections. A connected component is a set of nodes that are linked to each other node by paths, in this context, the giant component contains the most proportion of nodes of the network (Junker and Schreiber, 2011).

Centrality in a graph, *C*, measures the contributions or importance of a node in a network. As we are interested in the ranking of the node of the given graph *G*, we chose the convention that a node *u* is more important than another node *v* if *C(u) > C(v)*. The most relevant centrality metrics are: degree, closeness, betweenness and eigenvector centrality, which assigns every *v*∈*V* of a given graph *G* a value *C(v)*∈*R* (Junker and Schreiber, 2011).

In graph theory, a community is defined as a subset of nodes that can function independently. The Blondel’ algorithm assigns a different community to each node of the network, and then a node is moved to the community of one of its neighbors with which it achieves the highest positive contribution to modularity. This step is repeated for all nodes until no further improvement can be reached. Then, each community is considered as a single node on its own, and a subsequent step is repeated until there is only a single node left or when the modularity cannot be increased in a single step (Blondel et al., 2008). These metrics provide information about how connected the elements in a network and their module structure are (Junker and Schreiber, 2011; Lenz et al., 2020).

### Functional Annotation Analysis

To determine the biological process enriched in each community, we used the Database for Annotation, Visualization, and Integrated Discovery (DAVID 6.8^1^), which is a gene functional classification system that integrates a set of functional annotation tools (Jiao et al., 2012). Each list of genes from the communities were used to perform an enrichment analysis in Gene Ontology terms, a statistical significance at *P*-value of <0.05 was set (Supplementary Table 3).

### Cell Death Module

A total set of 430 genes related to cell death in *S. cerevisiae* was mined using the keywords: “cell death,” “apoptosis,” “autophagy,” and “necrosis,” in YEASTRACT+ (Monteiro et al., 2020). Those genes were also annotated with gene ontology and were considered as members of the cell death module in the GRN of *S. cerevisiae* (Supplementary Table 4). Based on this list, we identified by orthology criteria the cell death module within the GRN of *U. maydis* (Supplementary Table 4). This approach involves that the biological functions of each of the genes are conserved.

### *Ustilago maydis* Growth Conditions

*Ustilago maydis* wild-type strain FB2 (*a2b2*; donated by Banuett, 1992 California State University, Long Island, CA, United States) was maintained in 50% glycerol (v/v) at −70°C and recovered in liquid MC medium (0.5% peptone, 1% yeast extract, 1% glucose, 0.15% NO3NH4, 62.5 mL *U. maydis* salt solution l-1) (Holliday, 1961). Cells (1 × 106⋅mL-1) were inoculated into 500 ml of MC liquid medium and incubated at 28°C with shaking conditions at 180 rpm for 24 h (exponential phase), 48 h (stationary phase), and 72 h (loss of viability begins due to chronological aging) according to previous descriptions (Soberanes-Gutiérrez et al., 2020), and the samples were recovered by centrifugation. Cells were washed twice with sterile distilled water (SDW), collected by centrifugation at 1250 *g*, and used for ribonucleic acid (RNA) extraction.

### RNA Extraction and qRT-PCR Analysis

*Ustilago maydis* cells (1 × 10^6^⋅mL^–1^) were inoculated in liquid MC medium (Holliday, 1961) and incubated as described above. RNA was isolated from three independent cell cultures with Trizol reagent (Invitrogen, Carlsbad, CA, United States) following the manufacturer’s instructions. RNA isolated was treated with DNAse I (Invitrogen) and checked for yield and quality by measuring the absorbance ratio at A260/280 and A260/230 with a Nanodrop (Thermo Scientific, Waltham, MA, United States), and its integrity was observed by electrophoresis in denaturing agarose gels. First-strand cDNA was synthesized with SuperScript III Reverse Transcriptase (Invitrogen), using 5 mg RNA samples as the template. cDNA was quantified in a GeneQuant II spectrophotometer (Amersham Biosciences) and all samples containing the same quantity of first-strand cDNA (200 ng) were PCR-amplified per triplicate using KAPA SYBR FAST qPCR Master Mix (2X) kit with ROX (Kapa Biosystems, Merck KGaA, Darmstadt, Germany) according to the instructions of manufacturer and gene expression was quantified with a Step One Real-Time PCR system (Applied Biosystems, Foster City, CA, United States) with the oligonucleotides listed in Table 1.

**TABLE 1:** Sequences of forward and reverse primers used for qRT-PCR analysis.

**Name**	**Gene ID**	**Sequence (5′-3′)**
*rlm*1 Fwd	UMAG_10560	GCC GCC ATT CGT CAG AAG AGT
*rlm*1 Rev	UMAG_10560	CCT CTG GCA TTG CTG GAG AAG AC
*sin*3 Fwd	UMAG_11717	GCT CAA GCA GAA GGA CGA GGA G
*sin*3 Rev	UMAG_11717	GCA GGG TTG GAT CCA TGC TTA GTC
*aif*1 Fwd	UMAG_01967	GTG GAC CAA CGG GCG TAG AGT TC
*aif*1 Rev	UMAG_01967	GAG TGG TCC AGT TTG CTC TTG GTC
*tdh*3 (isoform A) Fwd	UMAG_02491 T0	GTC ATC CAC GAC AAG TTC GGT ATC G
*tdh*3 (isoform A) Rev	UMAG_02491 T0	GCA CAC GGA AAG CCA TAC CGG
*tdh3* (isoform B) Fwd	UMAG_02491 T1	CAG GTC GTC TCG AAC GCC TCA TG
*tdh3* (isoform B) Rev	UMAG_02491 T1	CGA GGG GAT GAT GTT GGC AGC G
*ald*4 Fwd	UMAG_05407 T1	GCAGACCTCCTCCAGCGAGAC
*ald*4 Rev	UMAG_05407 T1	CAG GAA CGT GCG AAG TGG TGC
*mca1* Fwd	UMAG_01408	GGACAGATAGAGGACGACGAGCTG
*mca1* Rev	UMAG_01408	GGC ATA GTT CAT GGC GGC ACC
*nuc*1 Fwd	UMAG_05674	GTACCAGCGAACAGCAAGCTCC
*nuc*1 Rev	UMAG_05674	GAG GTG TTC TGC GGT CCA TGA C
*ras1* Fwd	UMAG_00986	GCATCACATCTCGCAACTCTTTCG
*ras1* Rev	UMAG_00986	CGTTGATACGCTGCTTGGCTG
*atg8* Fwd	UMAG-10131	CATCTGCGAAAAGGCTGACC
*atg8* Rev	UMAG-10131	CTC GTC CTT GTG CTC TTC G
*tor1* Fwd	UMAG_00801	CGTTTGAGTATCGTCACAGCACC
*tor1* Rev	UMAG_00801	CGCGAACTGGGCTTGCATCAC
*act* Fwd	UMAG_11232	GACTTGACCGAGTACCTTGC
*act* Rev	UMAG_11232	CGTGATCACCTGTCCGTC

The actin gene was selected due to its stable expression and used as the housekeeping for qRT-PCR analysis. Threshold values (Ct) were used to quantify relative gene expression by the comparative 2–ΔΔCT method (Livak and Schmittgen, 2001). Expression levels shown in Log2 scale from relative quantification number (2–ΔΔCT). FB2 at 24 h was used as calibrator sample, therefore, ΔΔCT was obtained from the equation ΔCT(1T) – ΔCT (of each evaluated sample), where ΔCT represent [CT (selected gene)^∗^E]-[CT(Actin)^∗^E]. The efficiency (E) of each primer pair was evaluated using cDNAs as the template and was serially diluted 10-fold and the efficiency was determined from calibration curves using the formula ([10^(−1/slope)]−1). The thermocycling program consisted of one hold at 95°C for 1 min, followed by 39 cycles of 95°C for 15 s, 20 s at 60°C, and 20 s at 72°C. A melting-curve data was collected to verify specificity, contamination, and the absence of primer dimers. The samples were qRT-PCR amplified per triplicate, a blank (no template control) was also incorporated in each assay, and three independent experiments were performed.

### Statistical Analysis

The statistical analysis was performed using GraphPad Prism version 8 (GraphPad Software, California, United States). Unpaired *t*-test, One-Way ANOVA Analysis were performed to assess statistical significance. Data represent the mean ± SEM. *P*-values < 0.05 were considered as statistically significant.

## Results and Discussion

### Regulation in *U. maydis*

**FIGURE 1:**
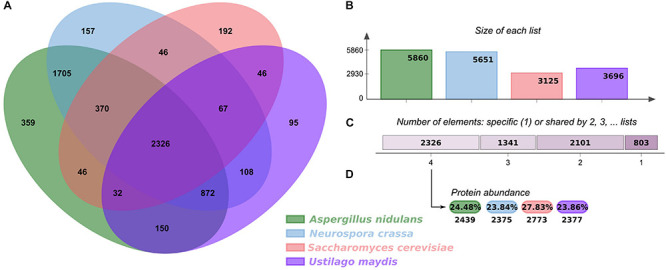
Orthologous proteins shared between *U. maydis, A. nidulans*, *N. crassa*, and *S. cerevisiae.* **(A)** Venn diagram comparing orthologous clusters of whole proteomes. **(B)** The bar plot graph shows the number of orthologous clusters by organism. **(C)** The plot indicates the number of clusters that are organism specific or shared by 2, 3, or 4 organisms. **(D)** For the 2326 clusters shared by 4 organisms, the protein abundance levels (in percentages and absolute numbers) are shown for each organism.

In order to analyze the regulatory elements in *U. maydis*, its GRN was reconstructed using as a reference three fungal models, *S. cerevisiae*, *A. nidulans*, and *N. crassa* (Hu et al., 2018; Monteiro et al., 2020). First, we analyzed the shared orthologous proteins between these four genomes and displayed them in OrthoVenn2 (Xu et al., 2019). The Venn diagram evidenced 2,326 clusters of 9,964 orthologous proteins common to all organisms, corresponding to 35.17% of the *U. maydis* proteome (Figure 1). The main functions associated with these proteins corresponded to rRNA processing (GO:0006364) (*P*-value: 2.638e-10) and translation (GO:0006412) (*P*-value: 2.353e-07) and are the most represented GO terms. This functional analysis indicates that core proteins shared by the four fungal species include those involved in the conversion of a primary ribosomal RNA (rRNA) into one or more mature rRNA molecules, as well as cellular metabolic process in which a protein is synthesized by using the sequence of a mature mRNA or circRNA molecule. The diagram also displays 95 clusters including 261 proteins specifically identified in *U. maydis*, suggesting that those proteins are species specific, and they represent 3.9% of genome proteins. These clusters include genes related to transcription DNA-template (GO:0006351), iron ion homeostasis (GO:0055072), response to wounding (GO:0009611), and hydrogen peroxide biosynthetic processes (GO:0050665).

An organism’s TF repertoire consists of a set of proteins that regulate gene expression in the cell. In fungi, approximately 80 families of TFs have been identified to date, and their proportions in genomes increase as genome size increases, with larger genomes having more TFs. However, the increase is mainly restricted to three major families: Zn_2_/Cys6 clusters, C_2_H_2_-like Zn fingers, and homeodomain-like (Shelest, 2017).

We compiled InterPro and PFAM predictions in *U. maydis*, and a set of 363 proteins described as TFs was identified that represent of 5.37% of proteome, these proteins are distributed in 59 families, where the most abundant are Zn2/Cys6 clusters (PF00172), which are comprised of 95 proteins (Table 2 and Supplementary Table 2). This domain is found in proteins that control a variety processes, such as carbon and nitrogen metabolism, amino acid and vitamin synthesis, stress response, pleiotropic drug resistance, meiosis and morphogenesis, among others (MacPherson et al., 2006), whereas the fungus-specific TF domain (PF04082) is associated with 41 proteins. This domain has been found in many fungal TFs involved in a wide diversity of cellular and metabolic processes (Ámon et al., 2017). Finally, C_2_H_2_-like Zn finger domain (PF00096) was found to be associated with 21 proteins. This domain is present in proteins related to gene transcription, translation, mRNA trafficking, protein folding and zinc sensing (Laity et al., 2001).

**TABLE 2:** Top 10 of the most abundant protein families in *U. maydis*.

**Pfam ID**	**Description**	**Total number of proteins**
PF00172	Zn_2_/Cys6 clusters domain	95
PF04082	Fungus-specific transcription factor domain	41
PF00096	C_2_H_2_-like Zn fingers domain	21
PF00010	Helix-loop-helix DNA-binding domain	13
PF00170	bZIP transcription factor	12
PF00642	Zinc finger C-x8-C-x5-C-x3-H type	12
PF00320	GATA zinc finger	11
PF00249	Myb-like DNA-binding domain	10
PS50249	STAS domain profile	8
PF00098	Zinc knuckle	7

### Gene Regulatory Network

The GRN in *U. maydis* was inferred from the orthology information and the GRNs of *A. nidulans*, *N. crassa* and *S. cerevisiae*. When orthologues of a TF-TG relationship in a model organism were identified for both TF and TG in *U. maydis*, a regulatory interaction was established (Yu et al., 2004; Galán-Vásquez et al., 2016).

**FIGURE 2:**
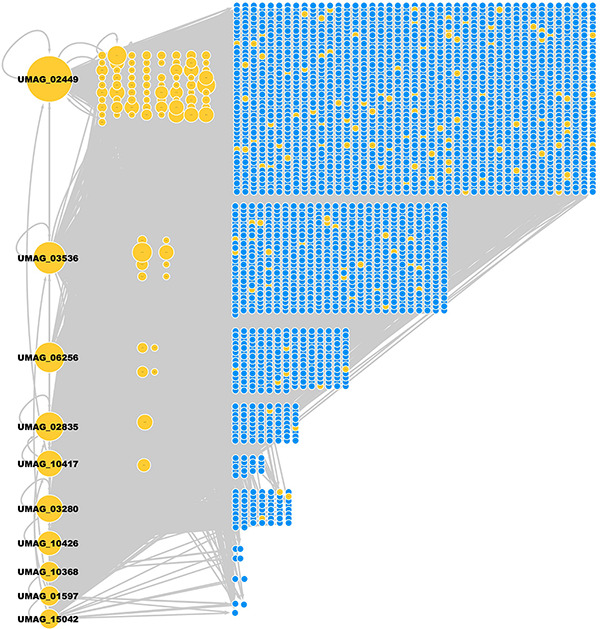
GRN of *U. maydis*. TFs (yellow nodes) and TGs (blue nodes). The most connected TFs (nodes) are UMAG_02449 (BHLH domain-containing protein), UMAG_03536 (hypothetical protein), UMAG_06256 (Zn(2)-C6 fungal-type domain-containing protein), UMAG_02835 (hypothetical protein), UMAG_10417 (hypothetical protein), UMAG_03280 (hypothetical protein), UMAG_10426 (pH-response TF pacC), UMAG_10368 (hypothetical protein), UMAG_01597 (Uncharacterized protein), and UMAG_15042 (Cell pattern formation-associated protein ust1).

The resulting network has 219 TFs, 2,849 TGs, and 23,932 regulatory interactions (Figure 2 and Supplementary Table 1) and covers 45.40% of the *U. maydis* proteome. The regulatory interactions inferred for *U. maydis* were preferentially assigned from *S. cerevisiae* (19,128 interactions), *N. crassa* (3,836 interactions), and *A. nidulans* (837 interactions).

From the 219 TFs in the GRN of *U. maydis*, 96 TFs regulate at least one TG inferred by homology and the rest of TFs are inferred by Interpro and Pfam. 50 TFs are self-regulated, *i.e.*, the TF regulates its own gene. In addition, three TFs (UMAG_02449, UMAG_03536, and UMAG_02835) regulate the highest number of additional TFs (158, 106, and 100 regulated TFs, respectively). UMAG_02357, UMAG_02835, UMAG_10417, and UMAG_05773 were the TFs most regulated by other TFs, with 21, 20, 20, and 20 regulatory TFs, respectively (Table 3).

**TABLE 3:** General properties of the regulatory network.

	**GRN of *U. maydis***
Total number of nodes	3068
Total number of interactions	23923
Number of TFs	219
Number of TGs	2849
Self-regulated	50
Maximum out degree	1997 (UMAG_02449)
Maximum in degree	43 (UMAG_10357)
Communities	17

In addition, UMAG_02449 (a BHLH domain-containing protein) is the TF that more genes regulate, with 1997 targets genes. This protein is homologous to the CBF1 protein which is necessary for chromosome segregation, and in response to DNA replication stress, protein abundance increases (Kent et al., 2004). On the other hand, the protein-gene encoding for UMAG_10357 (Branched-chain-amino-acid aminotransferase), homologous to BAT2, which is preferentially implicated in branched-chain amino acid catabolism (Colón et al., 2011), was found to be highly regulated by 43 different TFs.

### Topological Properties of the GRN

In order to characterize the global structure of the GRN of *U. maydis*, input degree (*Kin*) and output degree (*Kout*), which relate the number of TFs that regulate a gene and the number of genes that a TF regulates, respectively, were calculated. From this analysis, we observed that the maximum clustering coefficient is 1, indicating that nodes with neighbors who are related between them form complete graphs. This characteristic was found for 53 nodes (representing 1.43% of proteome), indicating that there are substructures such as triangles or more complex motifs. On the other hand, 508 nodes have a clustering coefficient equal to 0, which corresponds to 16.55% of nodes in the network; this is in part due to 496 of the nodes in the network having a degree of 1 and 2. We also found a mean 0.287 clustering coefficient for the network, indicating that neighbors have <1/3 connections among them. The nodes with highest clustering coefficient indicate that there are small highly connected groups, which may suggest the existence of a modularity in the network.

In addition, we identified the top 10 most important nodes by the metrics of the networks (Table 4). Centralities metrics are based on the nodes’ connectivity as well as the shortest paths between them. In this regard, the UMAG_02449 is the most significant node in terms of degree centrality (0.654). It codes for a putative TF-type basic helix-loop-helix, which is homologous to NCU08999 in *N. crassa*, AN7734 in *A. nidulans* and the protein CBF1 in *S. cerevisiae*, which is necessary for chromosome stabilization and methionine prototrophy. It is involved in chromosomal segregation and binds to CDEI, a closely conserved DNA sequence present in centromeres and multiple promoters (Peterson et al., 1998).

**TABLE 4:** Centralities of top 10 nodes.

**Level**	**Degree centrality**	**Closeness centrality**	**Betweenness centrality**	**Eigenvector centrality**
1	UMAG_02449	UMAG_10357	UMAG_05773	UMAG_10357
2	UMAG_03536	UMAG_11928	UMAG_02835	UMAG_01784
3	UMAG_06256	UMAG_01784	UMAG_06256	UMAG_11928
4	UMAG_02835	UMAG_11028	UMAG_02449	UMAG_06138
5	UMAG_03280	UMAG_04497	UMAG_04909	UMAG_11028
6	UMAG_10417	UMAG_04872	UMAG_10417	UMAG_03720
7	*UMAG_10426*	UMAG_06138	*UMAG_10426*	UMAG_04872
8	UMAG_10368	UMAG_01726	UMAG_01597	UMAG_04497
9	UMAG_01597	UMAG_02407	*UMAG_01224*	*UMAG_12244*
10	UMAG_15042	*UMAG_12244*	UMAG_03280	UMAG_04871

Furthermore, we identified that Putative branched-chain-amino-acid aminotransferase (UMAG_10357) is the node that minimizes the sum of distances to the other nodes, *i.e.*, the node with the high closeness score (0.017) as well, UMAG_10357 is the node that interacts with other highly connected nodes, *i.e*., the node with the highest eigenvector centrality (0.078). Thus, a protein has been described as a putative branched-chain-amino-acid transaminase that is homologous to BAT1 of *S. cerevisiae*, which is involved in both the biosynthesis and degradation of BCAAs, such as isoleucine, valine and leucine (Cherry et al., 2012).

UMAG_05773 was found to be the most significant when the betweenness centrality of a node *v* (0.0036) was calculated; it is determined as the sum of the fraction of all-pairs shortest paths passing through *v*, *i.e.*, the effect of a node on the information flow between any two nodes, assuming that this flow primarily along the shorts paths between them. UMAG_05773 is a hypothetical protein homologous to YMR136W of *S. cerevisiae*, that is, the GAT2 protein. It contains GATA family zinc finger motifs as Gln3p and Dal80p, and leucine inhibits expression (Forsberg et al., 2001; Table 4).

Finally, we identified that several proteins are in the top ten of more than one centrality, such as: UMAG_12244 which is homologous to SMB1 in *S. cerevisiae*, which encodes Small nuclear ribonucleoprotein-associated protein B; UMAG_11928 homologous to PCL1 in *S. cerevisiae*, which is a cyclin that regulates polarized growth and morphogenesis, as well as progression through the cell cycle; UMAG_10417, homologous to GAT1 in *S. cerevisiae*, which is a transcriptional activator of nitrogen catabolite repression gene; among others. Therefore, these proteins are important for monitoring and transmitting information within the network; *i.e*., they can be affected quickly by changes in any part of the network, and modulate expression changes in other parts of the network. At the structural level these proteins can connect the different subunits of the network.

We identified the top ten hubs in order to find the most connected TFs in the reconstructed network (Table 5). A hub was defined as a TF with connections with many other nodes. From these hubs, the two most connected are BHLH domain-containing protein (UMAG_02449), which regulates 1997 targets and is homologous to CBF1 in *S. cerevisiae* (see below) (Stoyan et al., 2001), and UMAG_03536, which regulates 1293 genes and is homologous to SFP1, which controls the transcription of ribosomal proteins and biogenesis genes, as well as the response to nutrients and stress, G2/M transitions during the mitotic cell cycle, the response to DNA damage, and modulates cell size (Marion et al., 2004). These 10 top hubs interact with 90% of the total target genes.

**TABLE 5:** Identified hubs in the reconstructed network.

**TFs**	**Locus ID**	**Number of TGs**	**Function**	**Homologous**	**References**
BHLH domain-containing protein	UMAG_02449	1997 genes	In *S. cerevisiae*, CBF-1 has the ability to bind to centromeric DNA elements I to ensure correct separation of chromosomes. In *N*. *crassa*, it is critical for circadian gene expression.	CBF1 in *S. cerevisiae*, NCU08999 in *N. crassa*, and AN7734 in *A. nidulans*	Stoyan et al., 2001; Cao et al., 2018
Transcription factor SFP1	UMAG_03536	1293 genes	Regulates ribosomal protein and biogenesis gene transcription and reaction to nutrients and stress, G2/M changes during the mitotic cell cycle, and DNA damage response, as well as cell size.	SFP1 of *S. cerevisiae*	Marion et al., 2004
Zn(2)-C6 fungal-type domain-containing protein	UMAG_06256	1196 genes	Positive transcription regulation from the RNA polymerase II promoter is involved in the cell’s response to chemical stimulus.	AN4558 in *A. nidulans*	Cerqueira et al., 2014
TEA domain-containing protein	UMAG_02835	1129 genes	Genes involved in hyphal growth, biofilm formation, and virulence are regulated by this TF.	TEC1 in *S. cerevisiae*	Schweizer et al., 2000
WD_REPEATS_REGION domain-containing protein	UMAG_03280	1047 genes	It is involved in the establishment of repressive chromatin structure through interactions with histones H3 and H4 and stabilization of nucleosomes over promoters.	TUP1 in *S. cerevisiae*	Williams et al., 1991
GATA-type domain-containing protein	UMAG_10417	1003 genes	Transcriptional activator of nitrogen catabolite repression genes.	GAT1 in *S. cerevisiae*	Kuruvilla et al., 2001
pH response transcription factor pacC/RIM101	UMAG_10426	896 genes	It is a Cys2His2 zinc-finger transcriptional repressor that is involved in alkaline responsive gene repression as part of the alkaline adaptation process.	RIM1 in *S. cerevisiae*	Lamb and Mitchell, 2003
HSF_DOMAIN domain-containing protein	UMAG_10368	690 genes	Activates several genes in response to a wide range of stresses; recognizes variable heat shock elements consisting of inverted NGAAN repeats.	HSF1 in *S. cerevisiae*	Bonner et al., 2000
Hypothetical protein	UMAG_01597	671 genes	Activator of transcription and global regulator of respiratory gene expression.	HAP2 in *S. cerevisiae*	Chodosh et al., 1988
Cell pattern formation-associated protein ust1	UMAG_15042	664 genes	Negatively regulates pseudohyphal differentiation and plays a regulatory role in the cyclic AMP (cAMP)-dependent protein kinase (PKA) signal transduction pathway.	SOK2 in *S. cerevisiae*	Pan and Heitman, 2000

### Identification of Communities in the Network

In order to identify the most related elements and uncover relations not previously described, we explored the network from the perspective of communities. A community is a subset of nodes that are densely connected as compared to the rest of the network (Radicchi et al., 2004). The *U. maydis* network contains 17 communities, where the longest contains 969 genes and the smallest contains 50 genes (Supplementary Table 3).

**FIGURE 3:**
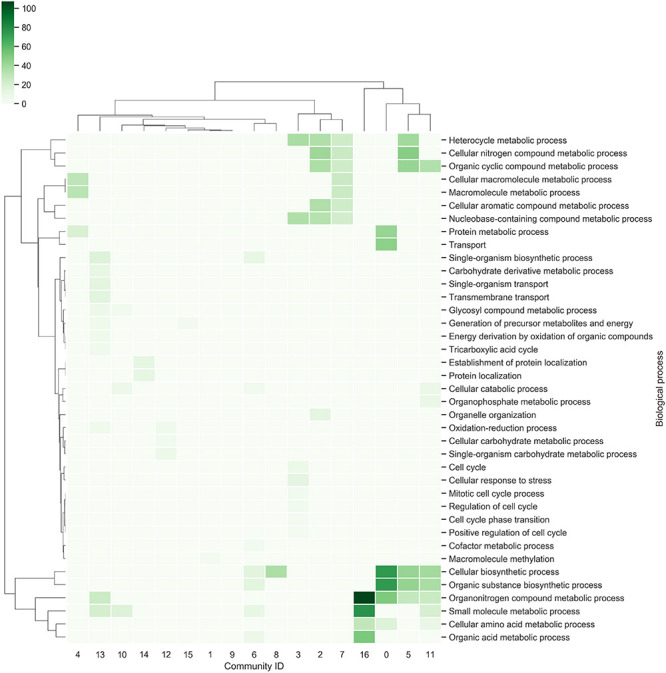
Communities in the network. The richest biological processes for each community were identified and hierarchically clustered based on Euclidean distance measures and Ward’s method for linkage analysis. Each row represents the GO term for biological processes, and each column represents the community ID.

Each community was functionally analyzed with the gene ontology (GO) terms enrichment (Figure 3). From this analysis, the communities with the greatest diversity of enriched biological processes are: Community-13, which contains genes related to the tricarboxylic acid cycle, energy derivation by oxidation of organic compounds and transmembrane transport, among others; Community-11, which contains genes related to cellular biosynthetic processes, organic substance biosynthetic processes, organonitrogen compound metabolic processes, among others; Community-3, which contains genes related to cellular response to stress, regulation of cell cycle, among others. On the other hand, the community-9 does not contain enriched biological processes, indicating that gene diversity is high (Figure 3).

### Module of Cell Death

**FIGURE 4:**
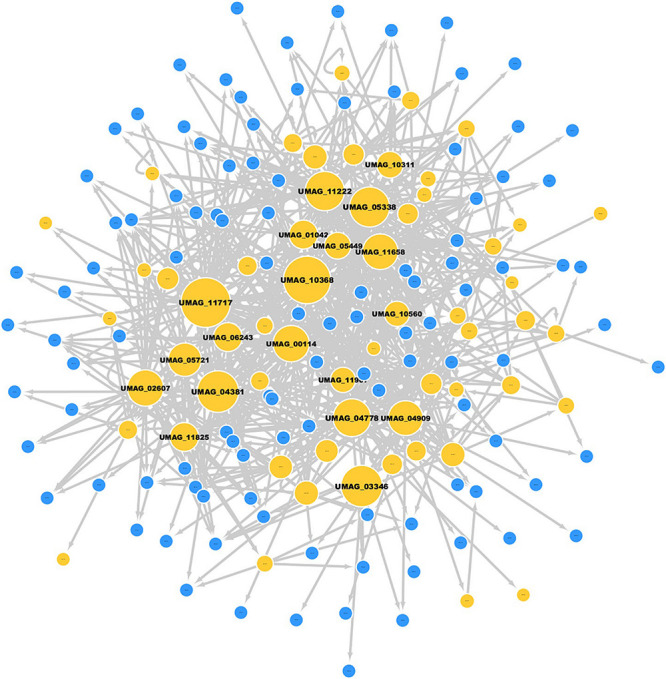
Cell death module in *U. maydis.* Nodes in yellow represent TFs, and nodes in blue are TGs; the gray edges represent regulatory interactions. The size of a node is proportional to its degree of output.

In order to study the regulation involved in viability in *U. maydis*, we identified a subset of 430 genes of *S. cerevisiae* involved in cell death, which included genes related to autophagy, apoptosis and necrosis (Cherry et al., 2012). We identified 168 orthologous genes involved in cell death, and this group is included in a module with 759 regulatory interactions in *U. maydis* (Figure 4 and Supplementary Table 4).

Topologically, the module consists of one giant component, with 60 TFs and 108 target genes, where the most highly connected node corresponds to the TF HDAC_interact domain-containing protein (UMAG_11717, which regulates 48 of the 168 target genes, including its own regulation). This protein is homologous to SIN3 of *S. cerevisiae*, which is associated with cell proliferation, energy metabolism, mating-type switching and meiosis, maintenance of chromosomal integrity and decrease cell death (Silverstein and Ekwall, 2005; Teng et al., 2011).

On the other hand, 14 different TFs regulate the expression of Glyceraldehyde-3-phosphate dehydrogenase (UMAG_02491) and UMAG_02491.1. Both TGs are the most regulated genes in the module and are homologous to TDH3 [isoform A] and TDH3 [isoform B] of *S. cerevisiae*, which are involved in glycolysis and gluconeogenesis (Delgado et al., 2001).

We also identified other genes of interest in the cell death module, such as the TF MADS-box domain-containing protein (UMAG_10560), which regulates 15 target genes and is the most central node based on eigenvector centrality (0.2333). This protein is involved in the maintenance of cell integrity (Jung et al., 2002). In addition, UMAG_05721 was identified as the most central node according to betweenness centrality (0.0629), and it is homologous to *srb1*, which encodes GDP-mannose pyrophosphorylase (mannose-1-phosphate guanyltransferase), an enzyme required for normal cell wall structure (Uccelletti et al., 2005). In the same context, UMAG_05687 is the most central node according to closeness centrality (0.1349); it encodes putative 20S proteasome subunit alpha 6, which is responsible for degradation of substrates of the ubiquitin pathways (Jung and Grune, 2013). Finally, we identified genes related to cell death previously studied in *U. maydis* and other organisms (Table 6).

**TABLE 6:** Genes related to cell death.

**Locus ID**	**Gene name**	**Number of TFs**	**Function**	**References**
UMAG_01408	MCA1 Metacaspase 1	6	This protein is needed for the clearance of insoluble proteins aggregates during normal growth and regulates apoptosis in response to H2O2 treatment.	Madeo et al., 2002; Mukherjee et al., 2017
UMAG_05674	NUC1 protein	4	It is a major mitochondrial nuclease that participates in mitochondrial recombination, apoptosis and maintenance of polyploidy.	Zassenhaus and Denniger, 1994
UMAG_00801	TORC1 protein	3	It is repressed by in presence of low concentrations of the metformin or curcumin it was down-regulated.	Soberanes-Gutiérrez et al., 2020
UMAG_00986	RAS1 protein	1	GTPase that participates in G-protein signaling in adenylate cyclase activation and regulates cell proliferation.	Tkach et al., 2012
UMAG_05567	ATG8 protein	10	It is a component of autophagosomes and Cvt vesicles.	Nair et al., 2012

### Gene Expression Analysis

To evaluate the impact of the genes related to cell death, we selected a set of 11 genes of interest from the cell death module identified. These included the most connected TFs in the cell death module (*sin3* or UMAG_11717), the most central node using eigenvector centrality (*rlm1* or UMAG_ 10560), the most regulated genes (*tdh3* [isoform A] or UMAG_02491, and *tdh3* [isoform B] or UMAG_02491.1), as well as, seven homologous genes involved in cell death in *S. cerevisiae* and *Candida albicans*: *mca1* (UMAG_01408), *nuc1* (UMAG_05674), *tor1* (UMAG_00801), *ras1* (UMAG_00986), *atg8* (UMAG_05567), *ald4* (UMAG_05407), and *aif1* (UMAG_01967). The expression levels of all the selected genes were measured by qRT-PCR, at 24 h, 48 h and 72, h. We chose the timepoints for qRT-PCR analysis according to previous reports (Soberanes-Gutiérrez et al., 2020): 24 h or exponential phase, at 48 h where the stationary phase begins, and at 72 h where it loses viability due to chronological aging in MC medium under our study conditions.

**FIGURE 5:**
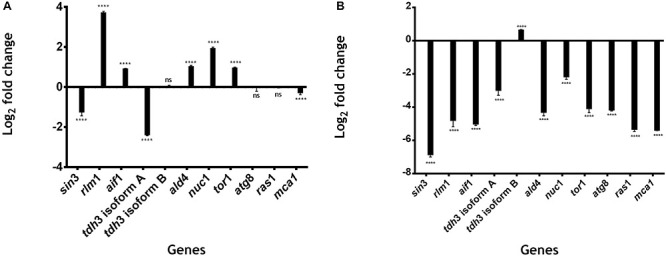
Gene expression related to cell death in *U. maydis.* 1 × 10^6^ cells⋅ml^–1^ were incubated in MC medium for 24 h (exponential phase), 48 h (stationary phase begins), and 72 h (aged yeast cells) and the expression of genes related to cell death was measured by qRT-PCR. **(A)** Log_2_ fold change in the expression of genes of *U. maydis* at 48 h of growth. **(B)** Log_2_ fold change in the expression of genes of *U. maydis* at 72 h of growth. The actin gene was used as an endogenous control, and the relative expression ratio of target genes was calculated by the 2^− ΔΔ*CT*^ method. All results are from three independent experiments. The values presented are the means ± SD for each group, ns is *P*-value > 0.05 and ***is *P*-value < 0.0001 vs. Control.

We identified five genes that are upregulated at 48 h: *rlm1* (3.734 log2-fold change); *aif1* (0.928 log2-fold change); *ald4* (1.039-log2-fold change); *nuc1* (1.95 log2-fold change); and *tor*1 (0.978 log2-fold change). All of these genes have significantly differentially expressions with *P* < 0.0001 *vs*. control 24 h (Figure 5A). *rlm1* is an ADS-box TF that is phosphorylated and activated by the MAP-kinase Slt2p and involved in cell integrity maintenance (Jung et al., 2002). *rlm1* is initially expressed to control stress conditions, and posteriorly it is then repressed; this is because cell death represses cell wall pathways (Jung et al., 2002). Three genes were downregulated: *sin3* (−1.26 log2-fold change), *tdh3* [isoform A] (−2.405 log2-fold change) and *mca*1 (−0.310 log2-fold change) with *P* < 0.0001 *vs*. control 24 h. *tdh*3 [isoform B], *atg*8 and *ras1* do not have a significantly differential expression at 48 h with a 0.049, −0.046 and −0.025 log2-fold change, respectively (Figure 5A).

On the other hand, 10 genes were downregulated at 72 h: *sin3* (−6.88 log2−fold change), *rlm1* (−4.815 log2−fold change), *aif1* (−5.039 log2−fold change), *tdh3* [isoform A] (−3.005 log2−fold change), *ald4* (−4.327 log2−fold change), *nuc1* (−2.196−log2−fold change), *tor1* (−4.111−log2-fold change), *atg8* (−4.197 log2-fold change), *ras*1(−0.025 log2-fold change), and *mca1* (−5.412 log2-fold change), with *P* < 0.0001 *vs*. control 24 h (Figure 5B). On the other hand, *tdh*3 [isoform B] was upregulated (0.662−log2−fold change) with *P* < 0.0001 *vs*. control 24 h (Figure 5B). All of these genes, except for *rlm1*, *ald4*, and *tdh3* [isoform A], are related to processes of programmed cell death, as SIN3, that is involved in the regulation of mitophagy receptor protein Atg32 in yeast (Aihara et al., 2014). ATG8 is an autophagic protein that plays a key role in autophagosome formation (Dufresne et al., 1998; Nadal and Gold, 2010; Khan et al., 2012); *mca1* plays a dual role in the induction of programmed cell death and intracellular protein quality control on exposure to stress conditions (Mukherjee et al., 2017); *aif1* is a conserved flavoprotein that causes chromatin condensation and DNA fragmentation, two hallmarks of apoptosis (Susin et al., 1999; Cande et al., 2004; Elguindy and Nakamaru-Ogiso, 2015; Ma et al., 2016); NUC1 causes apoptosis in yeast without the involvement of metacaspase or of apoptosis-inducing factor (Büttner et al., 2007); TOR kinase controls the initiation of autophagy, which results in the development of a single membrane structure known as the phagophore (Yorimitsu et al., 2007; Aguilar et al., 2017; Romero-Aguilar et al., 2020).

Interestingly the majority of genes was downregulated at 72 h, we hypothesize that the cell has a stage in which it makes use of protein turnover to obtain new amino acids, and inhibits gene expression in order to delay cell death. According to that, Herker et al. (2004) demonstrated that old yeast cells release substances into the medium that stimulate survival of the cells.

Additionally, ALD4 plays a critical role in the conversion of acetaldehyde to acetyl-CoA during growth on non-fermentable carbon sources and in the breakdown of toxic aldehydes that accumulate under stress conditions (Aranda and del Olmo, 2003). TDH3 is a Glyceraldehyde-3-phosphate dehydrogenase (GAPDH) involved in glycolysis and gluconeogenesis and catalyzes the reaction of glyceraldehyde-3-phosphate to 1,3 bis-phosphoglycerate (Delgado et al., 2001). GAPDH-derived antimicrobial peptides secreted by *S. cerevisiae* are active against a wide variety of wine-related yeasts and bacteria (Branco et al., 2014).

Finally, we observed that four genes, *aif1*, *ald4*, *tdh3* [isoform A] and *tdh3* [isoform B], do not have homologues in *S. reilianum*, which is the organism phylogenetically closest to *U. maydis*, these genes could be determinant in the greater cell viability shown by *S. reilianum* compared to *U. maydis* (Soberanes-Gutiérrez et al., 2020).

## Conclusion

Our approach of employing the GRN is a valuable resource of regulatory interactions in the general processes occurring in *U. maydis*. From this approach, proteins common to *U. maydis* and three fungal models were identified as involved in the conversion of a primary rRNA transcript into one or more mature rRNAs and in cellular metabolic processes. In addition, we found a large set of proteins devoted to regulating gene expression in this fungal system, where the Zn2/Cys6 clusters domain family (PF00172) is the most abundant, with 95 proteins. These proteins are involved in diverse metabolic processes, such as carbon and nitrogen, in the biosynthesis of amino acids and vitamins, pleiotropic drug resistance, stress response, and meiosis and morphogenesis, among others.

Concerning GRNs, we identified at least 10 global regulators, such as Helix-loop-helix domain-containing protein (UMAG_02449), protein homologous to CBF1 of *S. cerevisiae*, whose abundance increases in response to DNA replication stress. UMAG_03536, which regulates transcription of ribosomal and biogenesis genes and regulates responses to nutrients and stress, transitions during mitosis and response to DNA damage, among others; and Zn(2)-C6 fungal-type domain-containing protein (UMAG_06256), which is involved in transcription from RNApol II promoter associated to chemical stimulus.

Furthermore, in terms of communities, we evaluated the functional similarity of the genes clustered finding that genes related to organic nitrogen compound metabolic processes, cellular biosynthetic processes, and organic substance biosynthetic processes were the most abundant.

Additionally, we identified a regulatory module related to the cell death process that includes 60 TFs and 108 target genes. From this, HDAC_interact domain-containing protein is the most connected node; it is related to cell proliferation, energy metabolism, mating-type switching and meiosis, and maintenance of chromosomal integrity. This module includes genes previously reported as MCA1 metacaspase 1, NUC1, TORC1, and ATG8.

Finally, we analyzed a subset of nodes of the cell death module by qRT-PCR to study the expression levels of these genes at the beginning of the cell death process. We found that in the stationary phase (48 h), most of the genes previously reported to be involved in cell death did not have significant expression, while as cell death begins (72 h) these genes are down-regulated. Our results can serve as the basis for the study of transcriptional regulation, not only of the cell death process but also of all the cellular processes of *U. maydis*.

## Data Availability Statement

The original contributions presented in the study are included in the article/Supplementary Material, further inquiries can be directed to the corresponding author/s.

## Author Contributions

CS-G and EG-V: designed the study and carried out data acquisition and experiments. EP-R, CS-G, JR-H, and EG-V: drafted the manuscript and provided scientific advice and contributed to results interpretations. All authors read and approved the final manuscript.

## Conflict of Interest

The authors declare that the research was conducted in the absence of any commercial or financial relationships that could be construed as a potential conflict of interest.

## Footnotes

1 http://david.abcc.ncifcrf.gov/

## Supplementary Material

Supplementary Table 1 Gene regulatory network of *U. maydis* and *S. cerevisiae.*

Supplementary Table 2 Pfam domains in *U. maydis.*

Supplementary Table 3 Communities in GRN.

Supplementary Table 4 Module of cell death of *U. maydis*.
